# Wild Edible Plants of Rosoideae Subfamily: Correlation of Phenolic Content with Bioactivity

**DOI:** 10.3390/molecules31122026

**Published:** 2026-06-10

**Authors:** Serkos A. Haroutounian, Anna Apostolou, Lieve Naesens, Epameinondas Evergetis, Sandra Liekens, Eleni D. Myrtsi

**Affiliations:** 1 Laboratory of Nutritional Physiology & Feeding, Department of Animal Science, Agricultural University of Athens, Iera Odos 75, 11855 Athens, Greece; sehar@aua.gr (S.A.H.); annapost@msn.com (A.A.); 2 Rega Institute for Medical Research, KU Leuven, Herestraat 49, 3001 Leuven, Belgium; lieve.naesens@kuleuven.be (L.N.); sandra.liekens@kuleuven.be (S.L.)

**Keywords:** Rosoideae, antioxidant, antiproliferative, antiviral, phenols, influenza virus, H1N1, H3N2, adenovirus-2

## Abstract

Fruits of edible plants belonging to the Rosoideae subfamily are widely consumed as foods or utilized as herbs by various traditional medicine systems. Although these plants are mostly known for their rich phenolic content, there are only limited studies exploiting the relationship between their phenolic composition and bioactivities. The present study constitutes an exploratory chemical and bioactivity screening of fruits harvested from the following eight wildly grown edible Rosoideae plant species: *Rosa canina*, *Rosa sempervirens*, *Rosa pulverulenta*, *Rosa arvensis*, *Fragaria vesca*, *Rubus sanctus*, *Rubus idaeus* and *Sanguisorba officinalis*. In this context, the total phenolic and flavonoid contents of the investigated fruits were determined, and the presence of selected individual phenolic compounds was quantified. In addition, their antioxidant properties were evaluated by applying the ABTS and DPPH• assays, and their antiproliferative properties were assessed against selected tumor cell lines. Finally, the antiviral properties of fruits were investigated against fourteen common viruses. Respective results highlighted ellagic acid as the prevailing phenolic molecule for six investigated species, whereas several extracts displayed varying levels of antioxidant activities and moderate antiproliferative effects in the tested models. Furthermore, most extracts exhibited an inhibitory effect against Influenza viruses A (H1N1 and H3N2) and B, displaying IC_50_ values ranging from 0.6 to 4 μg/mL, comparable to currently used antiviral agents. Finally, the *Rubus idaeus* and *Rosa canina* fruit extracts were active against adenovirus-2. Since the bioactivities determined herein are based on single biological replicates, they are considered an indicative lead that provides an initial basis for prioritizing these edible Rosoideae species for future studies, which will involve a more detailed characterization of their bioactive phenolic constituents and more extensive, replicated biological experiments.

## 1. Introduction

Plants belonging to the Rosoideae subfamily of the Rosaceae family include several well-known edible species such as roses (*Rosa* spp.), strawberries (*Fragaria*) and raspberries (*Rubus*) that display a longstanding history and record of use as both foods and/or traditional medicines. Specifically, various roses have been appreciated since ancient Greek, Egyptian, and Persian times because of their fragrance, aesthetic appeal and ornamental values [1,2,3]. On the other hand, edible roses such as *Rosa rugosa* have extended their record of utilization as aromatic flowers, fruits or food ingredients in the form of edible flowers, since rose petals are utilized for the preparation of several foods and beverages, such as jams, teas and wines [4,5].

Besides their utility in food preparations, the role of Rosoideae plants in traditional medicine is also well documented [6,7,8]. In this context, many plants of this subfamily are incorporated into traditional medicines, frequently used for the treatment of various skin disorders like dermatitis and psoriasis or systemic health disorders such as diabetes and inflammation [9]. For example, *Rosa canina* is usually found in treatments of traditional Galician medicine [10] and *R. rugosa* is utilized to relieve liver and stomach pain in China. Finally, it is notable that the medicinal plants of the *Rosa* genus include at least ten widely studied medicinal plants displaying a long history of medicinal use [11].

It is evident that the intense bioactivity of Rosoideae subfamily plants is closely connected with their rich content of bioactive compounds such as flavonoids, tannins, phenolic acids, vitamins, and essential oils [12,13]. Since these compounds are well known to exhibit several beneficial health effects, they are inherently incorporated into medicinal preparations used in a wide range of pharmacological functions to afford antioxidant, anti-inflammatory, antimicrobial, antiviral, and cardiovascular system benefits [11]. Consequently, there are numerous studies concerning the antiviral properties of phenolic compounds, such as kaempferol and caffeic acid, which have been determined as potent inhibitors of dengue genome replication [14], or quercetin, which has the ability to suppress the viral growth cycles of several viruses [15]. The study herein aims to provide a comparative evaluation of the phenolic composition and biological activities of fruits obtained from eight edible Rosoideae subfamily plant species, namely *Rosa canina* (RC), *Rosa sempervirens* (RSe), *Rosa pulverulenta* (RP), *Rosa arvensis* (RA), *Fragaria vesca* (FV), *Rubus sanctus* (RuS), *Rubus idaeus* (RuI) and *Sanguisorba officinalis* (SO). The respective results indicate that their methanolic extracts are rich in phenolic compounds, with predominant amounts of ellagic acid, except for *R. canina* and *R. sanctus* species, which were found to contain high concentrations of rutin and gallic acid, respectively. The preliminary evaluation of their bioactivities revealed that they exhibit antioxidant, antiviral and, to a lesser extent, antiproliferative properties. Finally, it is noticeable that in terms of this preliminary study, all investigated extracts, apart from the *R. sanctus* species extract, exhibited potency against the Influenza A (H1N1 and H3N2) and B viruses, displaying IC_50_ values comparable to several currently used antiviral agents.

## 2. Results

The present endeavor constitutes an exploratory screening study designed to identify potentially bioactive edible Rosoideae species and provide an initial assessment of the possible relationship between their phenolic composition and bioactivities. The investigated samples consisted of fruits harvested from different parts of at least ten wildly grown individual plants and due to sample limitations, the bioactivity assessments were conducted as a single biological replicate per species. The determined activities, including IC_50_ values, should be regarded only as indicative observations rather than definitive quantitative measurements. Thus, the results presented herein provide preliminary evidence and guidance for future investigations involving expanded sampling and the implementation of various independent biological replicates along with a series of confirmatory biological studies.

### 2.1. Phenolic and Flavonoid Content

The determined values of total phenolic content (TPC) and total flavonoid content (TFC) are included in Table 1, where *F. vesca* is highlighted as the species with the highest phenolic content, followed by *S. officinalis* and *R. sempervirens*. With respect to the presence of flavonoids, *R. sempervirens* was identified as the richest source of flavonoids, with *R. pulverulenta* exhibiting a comparable flavonoid content. On the contrary, *R. sanctus* species was determined to display the lowest content for both classes of molecules. The quantitation of individual phenolic compounds present in the plant extracts investigated is included in Table 2, revealing the presence of thirteen bioactive phenols. Specifically, the following five species were determined to contain ellagic acid as the predominant phenolic compound: *Rosa pulverulenta* (1545.5 mg/100 g extract), *F. vesca* (1019.4 mg/100 g extract), *Rosa arvensis* (757.7 mg/100 g extract), *R. idaeus* (499.5 mg/100 g extract) and *S. officinalis* (452.6 mg/100 g extract). The molecule of ellagic acid, although detected at a low amount (18.0 mg/100 g extract), was also the prevailing phenolic molecule for the *R. sempervirens* species. On the other hand, no ellagic acid was detected in *R. canina* and *R. sanctus* fruits, which were found to contain the molecules of rutin and gallic acid in concentrations as the prevailing phenols at 123.4 and 381.6 mg/100 g for each extract, respectively.

### 2.2. Antioxidant Activity Results

**Figure 1:**
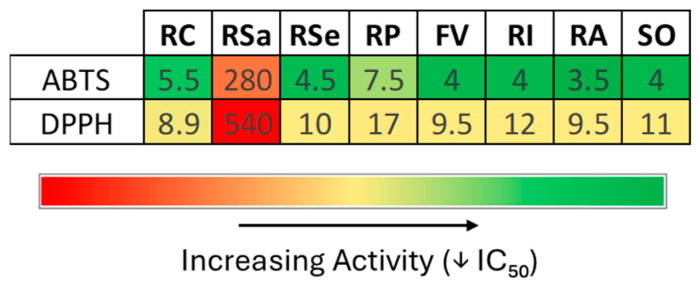
Representation of a heatmap for antioxidant activity of the methanolic extracts of the studied plants. Antioxidant activity was evaluated using ABTS and DPPH assays. Data are expressed as IC_50_ values (μg/mL). Green indicates increased activity (lower IC_50_), whereas red/beige indicates reduced activity (higher IC_50_).

The antioxidant capacities of the investigated plants were determined by applying the DPPH• and ABTS assays to their methanolic extracts. The detailed results, expressed as IC_50_ values, are provided as a heatmap in Figure 1. Specifically, the ABTS assay highlighted the *R. arvensis* species as exhibiting the highest antioxidant activity (IC_50_ 3.5 μg/mL), followed by *F. vesca*, *R. idaeus* and *S. officinalis*, which displayed IC_50_ values of 4 μg/mL. With respect to the DPPH assay, the most active species was determined to be *R. canina* (IC_50_ 8.9 μg/mL), followed by *F. vesca* and *R. arvensis*, both exhibiting IC_50_ values of 9.5 μg/mL. Evidently, *R. sanctus* species, which displayed the lowest TPC and TFC, was determined to be the least active in both assays.

### 2.3. Antiproliferative Properties Determination

**Figure 2:**
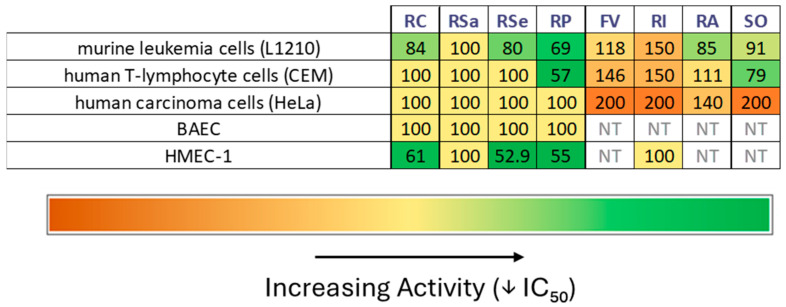
Representation of a heatmap showing the antiproliferative activity of methanolic extracts of the studied plants. Data are expressed as IC_50_ values (μg/mL). Green indicates increased activity (lower IC_50_), whereas red/beige indicates reduced activity (higher IC_50_). NT: Not tested.

The methanolic extracts of plant species studied were also investigated with respect to their antiproliferative activities against the following cell lines: murine leukemia cells (L1210), human T-lymphocyte cells (CEM), human carcinoma cells (HeLa), bovine aortic endothelial cells (BAEC) and microvascular endothelial cells (HMEC-1). The respective results are presented in Figure 2 as a heatmap and are included in Table S1 of the Supplementary Materials, revealing that most of the investigated fruits display moderate-to-low antiproliferative activity.

Nevertheless, the most active sample among the samples investigated was determined to be the methanolic extract of *R. pulverulenta*, which exhibited moderate activity against HMEC-1, human T-lymphocyte (CEM) and murine leukemia (L1210) cells, displaying IC_50_ values of 55, 57 and 69 μg/mL, respectively. Similarly, *R. canina* and *R. sempervirens* extracts also exhibited moderate activity with more prominent activity against the HMEC-1 cells, displaying IC_50_ values of 61 and 53 μg/mL respectively. Finally, since HMEC-1 cells correspond to immortalized human microvascular endothelial cells and have been included as a non-tumor endothelial model for a comparative evaluation of cytotoxicity, the activities observed in these results should not be interpreted as evidence of cancer-cell selectivity.

### 2.4. Antiviral Activities Assessment

**Figure 3:**
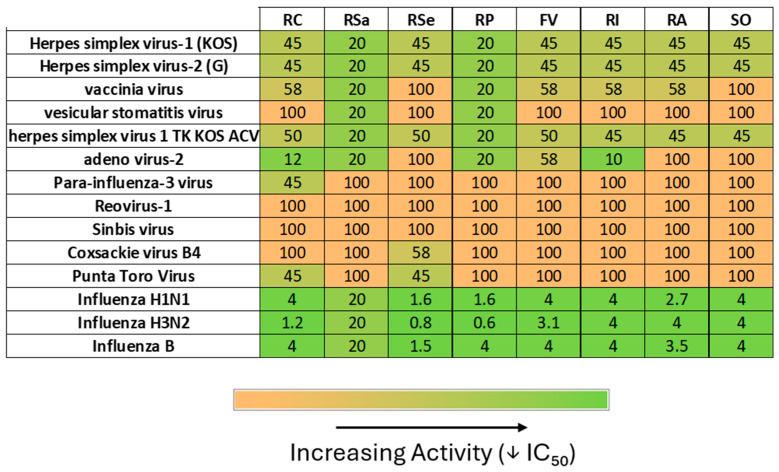
Representation of a heatmap showing antiviral activity of the methanolic extracts for the plants studied. Data are expressed as IC_50_ values (μg/mL). Green indicates increased activity (lower IC_50_), whereas red/beige indicates reduced activity (higher IC_50_).

The antiviral potency of the investigated plant species was screened against the following viruses: herpes simplex virus-1 (KOS), herpes simplex virus-2 (G), vaccinia virus, vesicular stomatitis virus, herpes simplex virus 1 TK KOS, adenovirus-2, Parainfluenza-3 virus, Reovirus-1, Sindbis virus, Coxsackie virus B4, Punta Toro Virus, as well as the Influenza viruses type A (H1N1 and H3N2) and type B. The respective results, along with the activities of currently used synthetic antiviral molecules, are presented as a heatmap in Figure 3 and included in Tables S2A, B and S3 of the Supplementary Materials section.

Seven out of eight plant extracts investigated were determined to exhibit pronounced antiviral effects against all studied Influenza viruses, whereas only *R. sanctus* displayed a comparatively lower activity. This preliminary observation may indicate a possible link between phenolic content and anti-influenza activity, as the *R. sanctus* extract was determined to possess the lowest phenolic content and the lowest antioxidant activity among all the samples investigated. As the most potent virus against Influenza A, the H3N2 virus was determined in the extracts of *R. pulverulenta* and *R. sempervirens* species, displaying IC_50_ values of 0.7 and 0.8 μg/mL, respectively. These values are comparable to those reported for the antiviral agents Amantadine and Zanamivir (0.7 and 1.1 μg/mL respectively), currently used and were more potent for Ribavirin (6.9 μg/mL). Accordingly, the same species also displayed the most intense activity against the Influenza A H1N1 virus, with IC_50_ values of 1.7 and 1.9 μg/mL for *R. pulverulenta* and *R. sempervirens*, respectively. The importance of these activities is evidenced by the fact that the respective values for Zanamivir, Amantadine and Ribavirin were 0.1, 4.4 and 8.5 μg/mL. Overall, the extracts of these two plant species were determined to exhibit a very potent activity against both strains of Influenza A virus. Finally, the extract of *R. sempervirens* was determined as the most active against the Influenza B virus, displaying an IC_50_ value of 1.6 μg/mL, followed by *R. pulverulenta* (3.6 μg/mL), while the respective values for Zanamivir, Ribavirin and Amantadine were 0.2, 7.7 and >200 μg/mL, respectively (Table S4).

With respect to the remaining viruses, the investigated plant extracts displayed moderate to weak antiviral activity against the eleven investigated strains, except the *R. sanctus* and *R. pulverulenta* extracts, which were determined to be active against six viruses. Finally, it is notable that the *R. canina* extract exhibited selective, potent activity against adenovirus-2, displaying an IC_50_ value of 12 μg/mL, which is comparable to the control molecule Cidofovir.

### 2.5. Multivariate Analysis of Polyphenolic Content and Bioactivity

**Figure 4:**
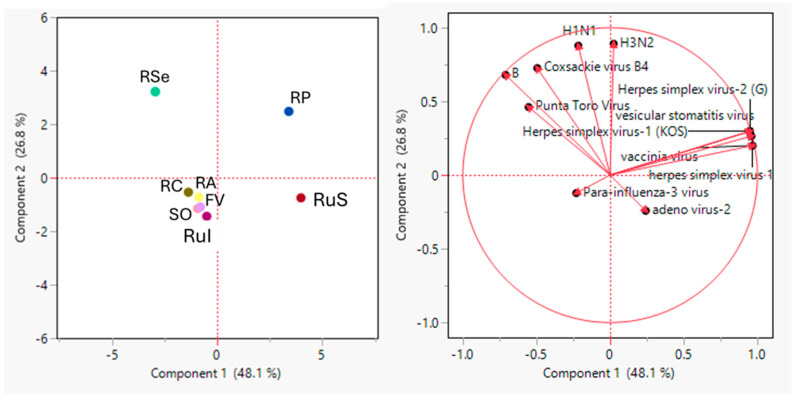
Principal Component Analysis (PCA) score plot based on antiviral activity data (expressed as activity = 1/IC_50_). PCA loading plot corresponding to the antiviral activity dataset.

**Figure 5:**
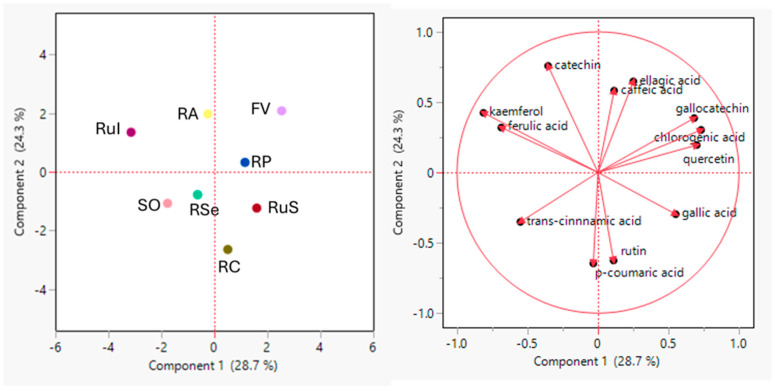
Principal Component Analysis PCA score plot based on the phenolic composition of the samples studied. PCA loading plot illustrates the contribution of individual phenolic compounds to sample differentiation.

Principal Component Analysis (PCA) was applied as an exploratory multivariate tool to visualize similarities and differences among the investigated plant extracts based on their antiviral activity profiles (Figure 4), as well as their chemical composition patterns (Figure 5). All statistical analyses were performed using the transformed activity values (activity = 1/IC_50_) rather than IC_50_, so that higher values correspond to higher biological activity. Given that only a single biological replicate per sample was analyzed, these findings are preliminary and should be interpreted as indicative trends instead of statistically conclusive evidence. Consequently, the observed clustering trends and correlations should be considered as indicative and not conclusive.

## 3. Discussion

Although the methanolic extract of *F. vesca* species was determined to display the highest TPC value and one of the most intense antioxidant capacities, it exhibited low antiproliferative and antiviral activities, except against Influenza viruses (H1N1, H3N2 and B), where it was determined to be very active, displaying IC_50_ values of 4 μg/mL for all strains. On the other hand, *R. sanctus,* the plant species exhibiting the lowest phenolic and flavonoid content along with the weakest antioxidant activity, displayed moderate antiviral activity against nine out of fourteen tested viruses. Specifically, the methanolic extract of *R. sanctus* was determined to be moderately active against herpes simplex virus-1 (KOS), herpes simplex virus-2 (G), vaccinia virus, vesicular stomatitis virus, herpes simplex virus 1 TK KOS ACV, adenovirus-2, and Influenza viruses H1N1, H3N2 and B, displaying an IC_50_ value of 20 μg/mL for each virus.

These findings reveal the well-established concept that the biological activity of plant extracts arises from synergistic interactions among multiple polyphenolic constituents rather than from a single dominant compound [16,17]. This interpretation is further supported by the PCA score plot constructed from the polyphenolic profiles of the samples, where no clear separation between phenolic groups was observed. Such overlapping distribution indicates that no individual class of compounds dominates the variability among samples, reinforcing the hypothesis that biological activity arises from synergistic interactions among multiple polyphenolic constituents (Figure 5). Importantly, seven out of eight investigated Rosoideae species extracts exhibited antiviral activity against Influenza virus strains A (H1N1, H3N2) and B. Among the tested samples, *R. pulverulenta* and *R. sempervirens* fruit extracts exhibited the lowest IC_50_ values against all investigated herein Influenza virus strains. Notably, the *R. pulverulenta* extract was also determined to contain a particularly high concentration of ellagic acid (1545.5 mg/100 g extract), followed by gallic acid (140.0 mg/100 g extract), while the *R. sempervirens* extract was relatively poor in these phenolic acids, containing 18.1 mg of ellagic and 2.4 mg of gallic acid/100 g of extract. Both ellagic and gallic acids are known to display significant antiviral properties against a wide range of viruses, since ellagic acid has been identified as a potent inhibitor of Ebola virus entry, acting early in the infection cycle before viral/cell membrane fusion [18]. In addition, it also exhibits moderate inhibitory activity against SARS-CoV-2 protease [19], while gallic acid displays significant anti-adenoviral activity by inhibiting the post-adsorption replication of viruses, thus enhancing its therapeutic potential [20].

Moreover, there are reports in the literature indicating that *Rosa* genus plants contain a variety of bioactive compounds such as flavonoids, terpenes, tannins and phenolic acids that contribute to various pharmacological effects, including antiviral activity [11]. However, studies concerning the antiviral properties of *R. pulverulenta* and *R. sempervirens* are not available. On the contrary, studies for other rose species, such as *Rosa damascena* and *Rosa alba*, indicate that their essential oils and extracts possess potent antiviral properties, particularly against herpes simplex virus type 1 (HSV-1) through the inhibition of viral adsorption, leading to the protection of cells from infection. These essential oils are also determined as capable of enhancing the efficacy of acyclovir against the resistant HSV-1 strains [21].

Regarding the PCA study illustrated in Figure 4 to assess the antiviral activities of samples, its outcome shows an evenly distributed variance between PC1 (48.1%) and PC2 (26.8%), reflecting a complex interaction between samples and viral inhibition. The dispersion of samples suggests that the antiviral effects determined are virus-dependent rather than governed by a single dominant factor. Specifically, regarding the separation of *R. pulverulenta* and *R. sanctus* along PC1, *R. pulverulenta* is indicative of stronger or distinct antiviral effects, while the *R. idaeus*, *S. officinalis* and *F. vesca* cluster, which appears on the negative side, suggests similar and possibly weaker activity profiles. Finally, samples positioned close to specific viral loading vectors, for example, the herpes simplex virus or adenovirus-2, indicate that they display selective antiviral effects. The above-mentioned observations explain that the separation of various species, such as *R. pulverulenta* and *R. sanctus*, alongside PC1 reflects differences in their virus-specific activity profiles.

On the other hand, *R. idaeus* and *R. canina* species extracts were determined to display an intense potency against adenovirus-2, displaying IC_50_ values of 10 and 12 μg/mL, respectively, followed by *R. sanctus* and *R. pulverulenta,* which both exhibited IC_50_ values of 20 μg/mL. Interestingly, these two extracts are in the same position at the antiviral PCA plots but in different positions in the polyphenol PCA plot, suggesting that their antiviral activity may be driven by different compounds. No previous reports on the *R. canina* antiviral activity are available in the literature, and there is only one study revealing that an aqueous–alcoholic extract of *R. idaeus* leaf displays strong inhibition activity against types three, five, and seven of the human adenovirus [22]. Thus, particular emphasis should be placed on the antiviral activity of these four extracts, considering that currently there is no specific drug formally approved against adenovirus-2. Nevertheless, although human adenoviruses usually cause mild and self-limited diseases, sometimes they can be severe, especially for children and immunocompromised or transplant patients [23,24]. Therefore, this study could serve as an initial step for a more thorough investigation of these extracts’ potential to act against adenoviruses.

Finally, regarding the anticancer properties investigated for the species studied, it must be noted that there are several literature reports indicating that extracts of several Rosa genus species are capable of inhibiting the growth of cancer cells. Indicatively, *Rosa rugosa* leaves are active against lung A549 cell lines [25] and *Rosa damascene* exhibits notable antiproliferative activity [26]. Furthermore, *R. canina* extracts have shown strong antiproliferative effects against colon cancer Caco-2 cells by modulating their redox status and inducing apoptosis [27]. In the present study, the methanolic extracts of *R. canina*, *R. sempervirens*, and *R. pulverulenta* were determined as active against murine leukemia L1210 and human microvascular endothelial HMEC-1 cells, displaying a moderate inhibitory effect against both cell lines, with IC_50_ values ranging from 69 to 84 μg/mL for L1210 and from 52.9 to 61 μM for HMEC-1. Notably, all three extracts exhibited slightly stronger activity against HMEC-1 cells, indicating a lack of cancer-cell selectivity. These findings suggest that the tested samples display a general cytotoxic activity and not a preferential antiproliferative activity against leukemia cells. However, their comparable or higher activity against endothelial cells may indicate a possible influence on vascular cell viability, which has yet to be investigated in terms of exploitation of their potential anti-angiogenic effects [28,29].

Finally, since it is well established that the phenolic composition and biological activity of plant extracts vary depending on environmental conditions, geographical origin, harvesting season, developmental stage, and extraction parameters, the absence of independent biological replicates for each investigated plant species constitutes an important limitation. Therefore, the findings reported herein should be considered preliminary and exploratory and future studies involving replicated sampling are necessary to confirm the observed trends.

## 4. Materials and Methods

### 4.1. Solvents, Chemicals and Standards

Analytical purity-grade methanol, ethanol, n-hexane and dimethylsulfoxide (DMSO) were obtained from Fisher Chemicals (Hampton, NH, USA). Water and acetonitrile were purchased as hypergrade LC–MS solvents from Merck (Darmstadt, Germany), while acetic acid and aluminum chloride hexahydrate were obtained from Sigma-Aldrich (Burlington, MA, USA). Anhydrous sodium carbonate, sodium hydroxide, potassium hydroxide (all >98%), sulfuric acid (98%), and hydrochloric acid were supplied by Chem-Lab (Zedelgem, Belgium). Ferric chloride hexahydrate, sodium nitrite and iron sulfate heptahydrate were supplied by Alfa Aesar (Heysham, UK).

Folin–Ciocalteu’s reagent, 2,2-diphenyl-1-picrylhydrazyl (DPPH), 2,2′-azino-bis(3-ethylbenzthiazoline-6-sulfonic acid) (ABTS), hydrogen peroxide (H_2_O_2_) and horseradish peroxidase (HRP) were purchased from Sigma-Aldrich (Burlington, MA, USA). All standards used for the determination of the contained phytochemicals were provided by Sigma-Aldrich (Burlington, MA, USA), except catechin and gallic acid, which were purchased from ExtraSynthese (Genay, France).

Finally, the PTFE filters (0.45 μm) were obtained from Macherey-Nagel, Duren, Germany.

### 4.2. Plant Material Sampling Extraction

Fresh fruits of the investigated Rosoideae subfamily plants were collected during the summer period (July–August) of 2022, from the respective wild plant colonies that grow in various mountains of Greece. The detailed collection data are included in Table S4 of the Supplementary Materials section, and each sample is composed of ten wildly grown plant fruits of the same species harvested from the respective collection site. After collection, fruits were mixed to provide 1 kg samples, which were analyzed and evaluated. A voucher specimen for each sampling material has been deposited in the herbarium of the Agricultural University of Athens.

In total, 500 g of each fruit sample was crushed, homogenized with a blender and lyophilized (Scientz-18N, Mujin Road, National Hi-tech Park, Ningbo, China). The resulting powder was placed in a conical flask containing 1 L of hexane and stirred in the dark for 48 h to remove the lipids and chlorophyll. The remaining solid was separated by filtration and placed in a conical flask. Then, 1 L of analytical purity methanol was added, and the resulting mixture was stirred in the dark for 48 h. The resulting solution was filtered and the solid was re-extracted twice following the same procedure. The combined methanolic extracts (3 L in total) were evaporated to dryness under reduced pressure and temperatures below 35 °C using a Büchi Rotavapor R-210 apparatus equipped with a Büchi vacuum pump V-700 and vacuum controller V-850 (all obtained from Büchi Labortechnik AG, Flawil, Switzerland), connected to a Julabo F12 cooling unit (Julabo GmbH, Seelbach, Germany). The resulting amorphous solid was ground into powder, which was stored in a freezer and used for analyses and determination.

### 4.3. Determination of Total Phenolic (TPC) and Flavonoid (TFC) Contents

The total phenolic content (TPC) of the extracts was evaluated by the Folin–Ciocalteu method as described previously by Singleton et al. [30]. Specifically, 100 μL of each sample was added to a 10 mL flask containing 6 mL of deionized water. One milliliter of Folin–Ciocalteu reagent was added to the mixture, and the flask was stoppered and allowed to stand at room temperature for 3 min. Then, 1.5 mL of 20% *w*/*v* sodium carbonate solution was added, and the solution was diluted to a 10 mL volume with deionized water. The mixture was incubated for 2 h at room temperature in the dark and the absorbance was measured on a spectrophotometer at 765 nm (Hitachi U-1900, Tokyo, Japan) against a blank containing the Folin–Ciocalteu reagent and distilled water. Each sample was measured in triplicate, and the mean value of the total phenolic content is expressed as mg of gallic acid equivalents (GAEs) per g of extract dried weight (dw).

The total flavonoid content (TFC) of samples was determined using a modified colorimetric method developed by Jia et al. [31]. Specifically, 1 mL of the methanolic extract was added to a 10 mL flask containing 4 mL of deionized water and 0.3 mL of sodium nitrite (5%) was added. The mixture was allowed to stand for 5 min at room temperature, before 0.3 mL of AlCl_3_·6H_2_O (10% ethanolic solution) was added and after 1 min, 2 mL of potassium hydroxide (1 M) was added. Then, the solution was diluted to 10 mL with the addition of deionized water; the absorbance was measured immediately at 510 nm. The value was calculated against a blank solution, and each sample was measured in triplicate. The mean value of the total flavonoid content is expressed as mg of quercetin equivalents (QEs) per g of the extract’s dry weight.

### 4.4. Determination and Quantitation of Presence of Individual Phytochemicals by HPLC and LC–MS/MS Analysis

The chemical composition of the extracts was determined by HPLC analysis, performed as previously described by Kerasioti et al. [30], using a Hewlett–Packard HP1100 instrument (Hewlett–Packard, Palo Alto, CA, USA), equipped with a quaternary pump, a degasser and an Agilent 1100 diode-array detector (DAD). This equipment was obtained from Agilent Technologies, Santa Clara, CA, USA. The system was coupled to ChemStation utilizing the manufacturer’s 5.01 software package system (Hewlett–Packard, Palo Alto, CA, USA). Chromatographic separation was achieved using a Zorbax Eclipse Plus C18, 5 μm, 150 × 4.6 mm i.d. chromatographic column (Agilent Technologies, Santa Clara, CA, USA), connected with a guard column of the same material (8 × 4 mm). Sample injection was performed using a Rheodyne injection valve (model 7725I) (Thermo Fischer Scientific, San Jose, CA, USA) with a 20 μL fixed loop. For the chromatographic analyses, HPLC-grade water was prepared using a Milli-Q system (Merck Millipore, Burlington, MA, USA), whereas all HPLC solvents (except acetonitrile) were filtered prior to use through cellulose acetate membranes of 0.45 μm pore size. The mobile phase was composed of a gradient system of 0.3% acetic acid in water (A) and acetonitrile (B). The flow rate was maintained at 1 mL/min and the column gradient elution program consisted of: 25% B (0 min), 25% B (5 min), 30% B (10 min), 40% B (15 min), 50% B (20 min), and 70% B (30 min). It was maintained for an additional 5 min, and returned over the course of 2 min to initial conditions, where it stayed for an additional 2 min. This routine was followed by a 15 min equilibration period with a zero-time mixture prior to injection of the next sample.

The quantification of phenolic compounds was performed by HPLC-DAD using external calibration curves constructed from reference standards at the characteristic wavelengths of each compound (280 nm: gallic acid, gallocatechin, catechin, syringic acid, ellagic acid, and trans-cinnamic acid; 320 nm: chlorogenic acid, caffeic acid, *p*-coumaric acid, and ferulic acid; 360 nm: quercetin, kaempferol, and rutin), while compound identity was confirmed by HPLC-MS/MS analysis operating in selected ion monitoring (SIM) and multiple reaction monitoring (MRM) modes. Confirmatory HPLC-MS/MS analysis was carried out on a Thermo Scientific Ultra-High-Performance Liquid Chromatography system coupled to a TSQ Quantum Vantage triple quadrupole mass spectrometer (Thermo Fischer Scientific, San Jose, CA, USA). Mass spectrometric analysis was conducted using heated electrospray ionization (HESI) operating in two complementary modes (positive and negative mode). The working conditions were as follows: spray voltage 4.2 kV and vaporizer and capillary temperatures of 280 and 260 °C respectively, while sheath and auxiliary gas corresponded to 60 and 40 arbitrary units. LC separation was achieved on a Hypersil Gold 3 μm. 150 × 3 mm i.d. chromatographic column (Thermo Fischer Scientific, San Jose, CA, USA). The mobile phase and the gradient system were identical to the above-mentioned for the HPLC analysis, using a flow rate of 0.3 mL/min [32].

### 4.5. Evaluation of Antioxidant Capacities

The antioxidant capacities of the investigated samples were estimated by performing the 2,2-DiPhenyl-1-PicrylHydrazyl (DPPH•) and the 2,2′-Azino-Bis(3-ethylbenzoThiazoline-6-Sulfonic acid) (ABTS•^+^) radical scavenging assays [33,34]. For this purpose, 200 mg of each sample was poured into 1 mL of methanol and stirred at room temperature for 20 min. Then, the supernatant was separated and used for the determination of its antioxidant capacity. Both assays were performed in triplicate, and the respective results were expressed as mean values ± standard deviations.

For the DPPH• assay, 1.0 mL of freshly made methanolic solution of DPPH• radical (100 μM) was mixed with the tested extract solution at different concentrations. The contents were vigorously mixed, incubated at room temperature in the dark for 20 min and the absorbance was measured at 517 nm using a Hitachi U-1900 ratio beam spectrophotometer (Hitachi, Tokyo, Japan). For each experiment, the pure sample was used as a blank and the DPPH• solution was used as a control.

The determination of radical scavenging with the ABTS•^+^ assay was performed in accordance with a previously described method with minor modifications [34]. Briefly, the ABTS•^+^ radical was produced by mixing 2 mM of ABTS with 30 μM H_2_O_2_ and 6 μM horseradish peroxidase (HRP) enzyme in 1 mL of distilled water. The solution was vigorously mixed and incubated for 45 min at room temperature in the absence of light to form the ABTS•^+^ radical. Then, 10 μL of various extract concentrations was added to the reaction mixture and the absorbance was measured at 730 nm using a Hitachi U-1900 ratio beam spectrophotometer (Tokyo, Japan). For each measurement, distilled water containing ABTS and H_2_O_2_ was used as a blank, and the ABTS•^+^ radical solution with 10 μL H_2_O was used as the control.

The percentage of radical scavenging capacity (RSC) of the tested extracts, for both assays, was calculated by applying the following equation:

Radical scavenging capacity (%) = [(Acontrol − Asample)/Acontrol] × 100

where Acontrol and Asample are the absorbance values of the control and the tested sample, respectively.

To compare the radical scavenging efficiency of extracts, the IC50 values recording the concentration causing 50% scavenging of DPPH• and ABTS• radicals were calculated from the graph plotted RSC percentage against extract concentration. The recorded IC50 values are expressed as μg/mL of extract solution and represent the mean value of experimental results carried out in triplicate.

### 4.6. Cytostatic Activity Assays

The cytostatic properties of the investigated plant extracts were evaluated by determining their potencies to inhibit the endothelial cell proliferation according to a previously published procedure [35]. Specifically, the inhibition of the following endothelial cells was investigated: murine leukemia cells (L1210), human T-lymphocyte cells (CEM), human carcinoma cells (HeLa), bovine aortic endothelial cells (BAECs) and microvascular endothelial cells (HMEC-1). All assays were performed by adding (5–7.5) × 10^4^ tumor cells to each well of 96-well microtiter plates along with a given amount of the test sample. The cells were allowed to proliferate for 48 h for L1210 or 72 h for human lymphocytic CEM and human cervix carcinoma HeLa cells at 37 °C in a humidified CO_2_-controlled atmosphere. At the end of the incubation period, the cells were counted and the inhibitory concentration (IC_50_), expressed as μg/g of dry extract, defined the concentration that inhibits the tumor cell proliferation by 50%.

### 4.7. Antiviral Properties Evaluation

The activity of the investigated plant extracts was evaluated against the following viruses: herpes simplex virus type 1 (HSV-1) strain KOS, herpes simplex virus type 2 strain G, vaccinia virus Lederle strain, vesicular stomatitis virus (VSV), herpes simplex virus type 1 (TK KOS ACV), adenovirus-2, Parainfluenza virus 3, Reovirus-1, Sindbis virus, Coxsackie virus B4, Punta Toro virus, Influenza virus A (subtypes H1N1, H3N2) and Influenza virus B. The antiviral assays were implemented in accordance with previously published procedures [35,36] by measuring the inhibition of virus-induced cytopathicity or plaque formation in human embryonic lung (HEL) fibroblasts, African green monkey cells (Vero), human epithelial cells (HeLa) or Madin–Darby canine kidney cells (MDCK). Confluent cell cultures in microtiter 96-well plates were inoculated with 100 CCID50 of the virus (with 1 CCID50 being the virus dose to infect 50% of the cell cultures) or with 20 or 100 plaque-forming units (PFU) (VZV or HCMV) in the presence of varying concentrations of the test compounds. Viral cytopathicity or plaque formation was recorded as soon as it reached completion in the control virus-infected cell cultures that were not treated with the test compounds.

Antiviral activity is expressed as the IC_50_ or compound concentration required to reduce virus-induced cytopathogenicity or viral plaque formation by 50%. For comparative purposes, the following reference antiviral agents were used as positive controls: Ribavirin, Amantadine, and Zanamivir for influenza viruses A and B, and Bivudin, Cidofovir, Acyclovir, Ganciclovir, DS-10.000 and Ribavirin for all remaining viruses.

### 4.8. Data Analysis

Statistical analyses were performed using Microsoft Excel 365 and JMP^®^ Student Edition (Edition 18). Calibration curves for the quantification of individual polyphenolic compounds were constructed using linear regression analysis in Microsoft Excel. Principal Component Analysis (PCA) was applied to explore similarities and differences among the plant extracts studied based on (i) their antiviral activity profiles and (ii) their polyphenolic composition. Prior to PCA, antiviral activity data were transformed to activity values (activity = 1/IC_50_) to ensure that higher values corresponded to higher biological activity and to facilitate comparison among samples. The datasets were mean-centered and scaled before analysis. PCA was performed using the correlation matrix to account for differences in variable magnitude. PCA score plots were used to visualize sample clustering patterns, while loading plots were examined to evaluate the contribution of individual variables (viruses or polyphenolic compounds) to sample differentiation. Because the present study was based on a single biological replicate per species, PCA was used as an exploratory multivariate tool to identify trends and relationships between chemical composition and antiviral activity rather than to establish statistically predictive models. Therefore, the results should be interpreted as indicative of potential associations that require confirmation through replicated experiments.

## 5. Conclusions

The present study provides a preliminary comparative phytochemical and biological evaluation of eight edible species classified into the Rosoideae subfamily and demonstrates that their methanolic extracts contain considerable amounts of phenolic compounds, exhibiting antioxidant and antiviral activities. Among the evaluated biological activities, the most notable observation concerns the antiviral activity recorded against Influenza A (H1N1 and H3N2) and Influenza B viruses, and across seven of the eight tested extracts. In addition, the extracts of *R. idaeus* and *R. canina* fruits displayed activity against adenovirus-2, suggesting that these species may represent interesting candidates for future antiviral investigations.

The antioxidant activity was correlated with total phenolic content, whereas the observed antiproliferative effects were moderate and did not indicate clear cancer-cell selectivity. Additionally, multivariate analysis suggested a possible relationship between phenolic composition and antiviral activity patterns. It must be noted, however, that these observations are exploratory, since PCA was performed using single biological replicate data. Although this work represents an exploratory screening study based on single biological replicates, the obtained results clearly identify *Rosa pulverulenta*, *Rubus sanctus*, and *Rosa canina* species as particularly promising candidates for further investigation and development. The outcomes of this initial screening study must be expanded to include investigations involving wider sampling and a bioactivity-guided fractionation to further explore the biological potential of edible Rosoideae species.

## Figures and Tables

**Table 1:** Total phenolic content (TPC) expressed as mg GAE/dried weight extract and total flavonoid content (TFC) expressed as mg QE/dried weight extract of studied plants.

Assays	*Rosa canina*	*Rubus sanctus*	*Rosa sempervirens*	*Rosa pulverulenta*	*Fragaria vesca*	*Rubus idaeus*	*Rosa arvensis*	*Sanguisorba officinalis*
TPC (mg GAE/g dw)	451.00	106.33	504.00	342.00	647.33	290.67	407.00	512.67
TFC (mg QE/g dw)	53.78	7.11	67.33	66.33	64.44	56.00	48.33	128.22

**Table 2:** Quantitation of presence of phenolic compounds in the plant species investigated expressed as mg/100 g of dried methanolic extract.

Compounds	*Rosa canina*	*Rubus sanctus*	*Rosa sempervirens*	*Rosa pulverulenta*	*Fragaria vesca*	*Rubus idaeus*	*Rosa arvensis*	*Sanguisorba officinalis*
catechin	ND	ND	ND	ND	22.1	23.7	26.9	17.9
rutin	ND	123.4	ND	ND	ND	ND	ND	ND
kaemferol	ND	ND	2.0	ND	ND	10.5	10.0	7.6
gallocatechin	ND	5.8	ND	15.5	27.9	ND	ND	ND
quercetin	39.7	25.6	ND	12.3	38.8	2.9	39.2	9.5
ellagic acid	ND	ND	18.1	1545.5	1019.4	499.5	757.7	452.6
gallic acid	381.6	98.5	2.4	140.0	63.1	17.6	52.7	11.8
chlorogenic acid	71.4	10.4	ND	ND	101.9	ND	23.1	ND
caffeic acid	8.6	4.5	1.8	9.8	6.3	10.5	11.5	ND
*p*-coumaric acid	4.9	7.7	ND	5.9	1.1	2.3	1.9	8.4
ferulic acid	ND	ND	ND	ND	ND	8.9	ND	ND
*trans*-cinnamic acid	6.4	3.8	ND	ND	ND	7.9	ND	7.4

ND: Not detected.

## Data Availability

The original contributions presented in this study are included in the article. Further inquiries can be directed to the corresponding author.

## Supplementary Materials

The following supporting information can be downloaded at https://www.mdpi.com/article/10.3390/molecules31122026/s1. Table S1. Antiproliferative activities οf investigated Rosoideae plant methanolic extracts. Data are expressed as IC_50_ values (μg/mL). Table S2A. Antiviral activity of methanolic extracts of studied plants. Data are expressed as IC_50_ values (μg/mL). Table S2B. Antiviral activity of methanolic extracts of studied plants. Data are expressed as IC_50_ values (μg/mL). Table S3. Methanolic extracts activities of studied plants against Influenza A (H1N1, H3N2) and B. Data are expressed as IC_50_ values (μg/mL). Table S4. Plant material sampling data.

## References

[1] Marchioni I., Pistelli L., Copetta A., Dimita R., Descamps S., Cambournac L., Ruffoni B. (2021). Edible Roses as Novel Food with Healthy Value. Acta Hortic..

[2] Çiçek H., Kaya H.S., Kilic C., Savas M., Ravichandran S. (2022). Medicinal Effects of Products Obtained from Wild Rose Plant. J. Chem. Nutr. Biochem..

[3] Meng Q., Manghwar H., Hu W. (2022). Study on Supergenus *Rubus* L.: Edible, Medicinal, and Phylogenetic Characterization. Plants.

[4] Hegde A.S., Gupta S., Sharma S., Srivatsan V., Kumari P. (2022). Edible Rose Flowers: A Doorway to Gastronomic and Nutraceutical Research. Food Res. Int..

[5] Tursun X., Zhao Y., Alat Z., Xin X., Tursun A., Abdulla R., AkberAisa H. (2016). Anti-Inflammatory Effect of *Rosa rugosa* Flower Extract in Lipopolysaccharide-Stimulated RAW264.7 Macrophages. Biomol. Ther..

[6] Mileva M., Ilieva Y., Jovtchev G., Gateva S., Zaharieva M.M., Georgieva A., Dimitrova L., Dobreva A., Angelova T., Vilhelmova-Ilieva N. (2021). Rose Flowers—A Delicate Perfume or a Natural Healer?. Biomolecules.

[7] Patel A., Rojas-Vera J., Dacke C. (2004). Therapeutic Constituents and Actions of *Rubus* Species. Curr. Med. Chem..

[8] Gevrenova R., Zheleva-Dimitrova D., Balabanova V. (2024). The Genus *Rubus* L.: An Insight into Phytochemicals and Pharmacological Studies of Leaves from the Most Promising Species. Pharmacia.

[9] Cristea A.M., Smeu A., Cîmpeanu I.-A., Iftode A., Liga S., Tchiakpe-Antal D.-S., Vlad D., Dehelean C.A., Iliescu D. (2025). Biological Effects of Rosaceae Species in Skin Disorders—An Up-To-Date Overview. Plants.

[10] Garcia-Oliveira P., Fraga-Corral M., Pereira A.G., Lourenço-Lopes C., Jimenez-Lopez C., Prieto M.A., Simal-Gandara J. (2020). Scientific Basis for the Industrialization of Traditionally Used Plants of the Rosaceae Family. Food Chem..

[11] Wang Y., Zhao Y., Liu X., Li J., Zhang J., Liu D. (2022). Chemical Constituents and Pharmacological Activities of Medicinal Plants from Rosa Genus. Chin. Herb. Med..

[12] Simin N., Lesjak M., Živanović N., Božanić Tanjga B., Orčić D., Ljubojević M. (2023). Morphological Characters, Phytochemical Profile and Biological Activities of Novel Garden Roses Edible Cultivars. Horticulturae.

[13] Demasi S., Caser M., Donno D., Enri S.R., Lonati M., Scariot V. (2021). Exploring Wild Edible Flowers as a Source of Bioactive Compounds: New Perspectives in Horticulture. Folia Hortic..

[14] Kyriakopoulou E., Tsakni A., Korakidis E., Mpekoulis G., Kalliampakou K.I., Polanska M., Van Impe J.F.M., Tsakali E., Houhoula D., Vassilaki N. (2025). Evaluation of Polyphenolic Compounds Common in Greek Medicinal Plants for Their Antioxidant Effects and Antiviral Activity Against Dengue and Yellow Fever Viruses. Antioxidants.

[15] Chojnacka K., Skrzypczak D., Izydorczyk G., Mikula K., Szopa D., Witek-Krowiak A. (2021). Antiviral Properties of Polyphenols from Plants. Foods.

[16] Wink M. (2022). Current Understanding of Modes of Action of Multicomponent Bioactive Phytochemicals: Potential for Nutraceuticals and Antimicrobials. Annu. Rev. Food Sci. Technol..

[17] Brglez Mojzer E., Knez Hrnčič M., Škerget M., Knez Ž., Bren U. (2016). Polyphenols: Extraction Methods, Antioxidative Action, Bioavailability and Anticarcinogenic Effects. Molecules.

[18] Cui Q., Du R., Anantpadma M., Schafer A., Hou L., Tian J., Davey R., Cheng H., Rong L. (2018). Identification of Ellagic Acid from Plant *Rhodiola rosea* L. as an Anti-Ebola Virus Entry Inhibitor. Viruses.

[19] Navarro F., Hamri S., Reches R., Viñas M., Jahani D., Ginard J., Vilardell J., Abián O., Pujol M.D. (2023). Convenient Synthesis of Ellagic Acid from Methyl Gallate and SARS-CoV-2 3CLpro Antiviral Activity. Synthesis.

[20] Karimi A., Moradi M.-T., Rabiei M., Alidadi S. (2020). In Vitro Anti-Adenoviral Activities of Ethanol Extract, Fractions, and Main Phenolic Compounds of Pomegranate (*Punica granatum* L.) Peel. Antivir. Chem. Chemother..

[21] Vilhelmova-Ilieva N., Dobreva A., Doynovska R., Krastev D., Mileva M. (2021). Antiviral Activity of *Rosa damascena* Mill. and *Rosa alba* L. Essential Oils against the Multiplication of Herpes Simplex Virus Type 1 Strains Sensitive and Resistant to Acyclovir. Biology.

[22] Povnitsa O.Y., Artiukh L., Pankivska Y., Likhanov A., Doroskyh A., Lysenko V., Lokshyn M., Zahorodnia S.D. (2021). In Vitro Antiviral Activity of Leaf Extracts Plantago Major, Plantago Lanceolata, *Rubus* Idaeus. Mikrobiolohichnyi Zhurnal.

[23] Dodge M.J., MacNeil K.M., Tessier T.M., Weinberg J.B., Mymryk J.S. (2021). Emerging Antiviral Therapeutics for Human Adenovirus Infection: Recent Developments and Novel Strategies. Antivir. Res..

[24] Rübsamen-Schaeff H., Buschmann H. (2022). New Drug Development for Known and Emerging Viruses. Methods and Principles in Medicinal Chemistry.

[25] Kim E., Mok H.K., Hyun T.K. (2022). Variations in the Antioxidant, Anticancer, and Anti-Inflammatory Properties of Different *Rosa rugosa* Organ Extracts. Agronomy.

[26] Faur C.-A., Zăhan M., Bunea C.I., Hârșan E., Bora F.-D., Bunea A. (2024). Antiproliferative and Biochemical Evaluation of Rose Extracts: Impact on Tumor and Normal Skin Cells. Front. Plant Sci..

[27] Jiménez S., Gascón S., Luquin A., Laguna M., Ancin-Azpilicueta C., Rodríguez-Yoldi M.J. (2016). Rosa Canina Extracts Have Antiproliferative and Antioxidant Effects on Caco-2 Human Colon Cancer. PLoS ONE.

[28] Choi B.-K., Jo M.-H., Shin H.J., Park S.J. (2025). Anti-Angiogenic Potential of Marine Streptomyces-Derived Lucknolide A on VEGF/VEGFR2 Signaling in Human Endothelial Cells. Molecules.

[29] Oliveira R.F.D., Stoltz I.R., Gonçalves P.G., Echevarria A., Taborda L., Lepinsk Lopes R.H., Pereira L.F., Elifio-Esposito S. (2024). Evaluation of the Antitumoral Effects of the Mesoionic Compound MI-D: Implications for Endothelial Cells Viability and Angiogenesis Inhibition. Chem.-Biol. Interact..

[30] Singleton V.L., Orthofer R., Lamuela-Raventós R.M. (1999). Analysis of Total Phenols and Other Oxidation Substrates and Antioxidants by Means of Folin-Ciocalteu Reagent. Methods in Enzymology.

[31] Zhishen J., Mengcheng T., Jianming W. (1999). The Determination of Flavonoid Contents in Mulberry and Their Scavenging Effects on Superoxide Radicals. Food Chem..

[32] Kerasioti E., Apostolou A., Kafantaris I., Chronis K., Kokka E., Dimitriadou C., Tzanetou E.N., Priftis A., Koulocheri S.D., Haroutounian S.A. (2019). Polyphenolic Composition of Rosa Canina, Rosa Sempervivens and Pyrocantha Coccinea Extracts and Assessment of Their Antioxidant Activity in Human Endothelial Cells. Antioxidants.

[33] Brand-Williams W., Cuvelier M.E., Berset C. (1995). Use of a Free Radical Method to Evaluate Antioxidant Activity. LWT-Food Sci. Technol..

[34] Cano A., Hernández-Ruíz J., García-Cánovas F., Acosta M., Arnao M.B. (1998). An End-Point Method for Estimation of the Total Antioxidant Activity in Plant Material. Phytochem. Anal..

[35] Dinh Ngoc T., Moons N., Kim Y., De Borggraeve W., Mashentseva A., Andrei G., Snoeck R., Balzarini J., Dehaen W. (2014). Synthesis of Triterpenoid Triazine Derivatives from Allobetulone and Betulonic Acid with Biological Activities. Bioorganic Med. Chem..

[36] Pertusati F., Hinsinger K., Flynn Á.S., Powell N., Tristram A., Balzarini J., McGuigan C. (2014). PMPA and PMEA Prodrugs for the Treatment of HIV Infections and Human Papillomavirus (HPV) Associated Neoplasia and Cancer. Eur. J. Med. Chem..

