# Supplementary material for: Wild Edible Plants of Rosoideae Subfamily: Correlation of Phenolic Content with Bioactivity
Source: Molecules. 2026 Jun 10;31(12):2026. doi: 10.3390/molecules31122026 (PMC13304982; doi:10.3390/molecules31122026)
Supplement: Supplementary file 1 [file molecules-31-02026-s001.zip › molecules-4333871-supplementary.pdf]

Supplementary Table S1. Antiproliferative activities of investigated Rosoideae plant methanolic extracts. Data are expressed as IC_{50} values (µg/mL).

**Cell Lines**	***Rubus sanctus***	***Rosa canina***	***Rosa sempervirens***	***Rosa pulverulenta***	***Fragaria vesca***	***Rubus idaeus***	***Rosa arvensis***	***Sanguisorba officinalis***
Murine leukemia cells (L1210)	100	84	80	69	118	150	85	91
Human T-lymphocyte cells (CEM)	100	100	100	57	146	150	111	79
Human carcinoma cells (HeLa)	100	100	100	100	200	200	140	200
Bovine Aortic Endothelial Cells (BAEC)	100	100	100	100	NT[1]	NT	NT	NT
Human dermal Microvascular Endothelial Cells (HMEC-1)	100	61	53	55	NT	100	NT	NT

Supplementary Table S2-A. Antiviral activity of methanolic extracts of studied plants. Data are expressed as IC_{50} values (µg/mL).

Taxa	*Rubus sanctus*	*Rosa canina*	*Rosa sempervirens*	*Rosa pulverulenta*	*Fragaria vesca*	*Rubus idaeus*	*Rosa arvensis*	*Sanguisorba officinalis*	Bivudin	Cidofovir	Acyclovir	Ganciclovir
Herpes simplex virus-1 (KOS)	20	45	45	20	45	45	45	45	0.05	1.5	0.4	0.03
Herpes simplex virus-2G	20	45	45	20	45	45	45	45	250	2	0.4	0.03
Vaccinia virus	20	58	100	20	60	58	60	100	25	22	>250	>100

Vesicular stomatitis virus	20	100	100	20	100	100	100	100	>250	>250	>250	>100
Herpes simplex virus-1 TK (KOS)	20	50	50	20	50	45	45	45	10	2	5.9	0.8
Adenovirus-2	20	12	100	20	58	10	100	100	-	10	-	-

Supplementary Table S2-B. Antiviral activity of methanolic extracts of studied plants. Data are expressed as IC_{50} values (µg/mL).

Taxa	*Rubus sanctus*	*Rosa canina*	*Rosa sempervirens*	*Rosa pulverulenta*	*Fragaria vesca*	*Rubus idaeus*	*Rosa arvensis*	*Sanguisorba officinalis*	DS-10.000	Ribavirin
Parainfluenza-3 virus	100	45	100	100	100	100	100	100	>100	146
Reovirus-1	100	100	100	100	100	100	100	100	>100	112
Sindbis virus	100	100	100	100	100	100	100	100	100	>250
Coxsackie virus B4	100	100	58	100	100	100	100	100	34	112

Punta Toro virus	100	45	45	100	100	100	100	100	100	112

Supplementary Table S3. Methanolic extracts activities of studied plants against influenza A (H1N1, H3N2) and B. Data are expressed as IC_{50} values (μg/mL).

Taxa	*Rosa canina*	*Rubus sanctus*	*Rosa sempervirens*	*Rosa pulverulenta*	*Fragaria vesca*	*Rubus idaeus*	*Rosa arvensis*	*Sanguisorba officinalis*	Zanamivir	Ribavirin	Amantadine
Influenza A H1N1	4	20	1.9	1.7	4	4	3.9	4	0.1	8.5	4.4
Influenza A H3N2	1.3	20	0.8	0.7	4	4	4	4	1.1	6.9	0.7
Influenza B	4	20	1.6	3.6	4	4	3.8	4	0.2	7.7	>200

Supplementary Table S4. Plant material sampling data.

Code	Taxa	Location	Altitude	Code
RC	*Rosa canina*	Mt. Parnitha	1013	N 38o08.939' E 023o43.171'
RuS	*Rubus sanctus*	Mt. Giona	948	N 38o39.054' E 022o22.955'
RSe	*Rosa sempervirens*	Mt. Parnitha	1047	N 38o09.413' E 023o43.683'
RP	*Rosa pulverulenta*	Mt. Giona	1360	N 38o40.141' E 022o18.510'
FV	*Fragaria vesca*	Mt. Tymfi	1018	N 39o58.305' E 020o43.883'
RuI	*Rubus idaeus*	Mt Grammos	1044	N 40o23.392' E 020o83.289'
RA	*Rosa arvensis*	Mt. Giona	1308	N 38o40.410' E 022o17.686'
SO	*Sanguisorba officinali*	Mt Tymfi	1083	N 39o59.199' E 020o45.392′
